# Efficacy and safety of HIMABERB® Berberine on glycemic control in patients with prediabetes: double-blind, placebo-controlled, and randomized pilot trial

**DOI:** 10.1186/s12902-023-01442-y

**Published:** 2023-09-07

**Authors:** Antarmayee Panigrahi, Susant Mohanty

**Affiliations:** Department of Pedodontics and Preventive Dentistry, Institute of Dental Sciences, Siksha O Anusandhan (Deemed to be University), Bhubaneshwar, 751030 Odisha India

**Keywords:** Berberine, Prediabetes, Glycemic control, Insulin resistance, HIMABERB®

## Abstract

**Background:**

Prediabetes and diabetes involve alterations in glucose homeostasis, including increased fasting blood glucose and impaired glucose tolerance. Berberine has been identified as a potential regulator of glucose homeostasis with implications on the management of type 2 diabetes mellitus (DM). Given a paucity of data on berberine in prediabetes, evaluation of its effect in individuals with prediabetes may prove clinically valuable.

**Objective:**

The present pilot study aimed to investigate the effect of daily oral berberine on markers of glycemic control and insulin resistance among individuals with prediabetes.

**Methods:**

A randomized, double-blinded, placebo-controlled trial was conducted for 12 weeks among 34 individuals with prediabetes as defined by the American Diabetes Association (fasting plasma glucose (FPG) between 5.6 and 6.9 mmol/L, glycosylated hemoglobin (HbA_1c_) between 5.7% and 6.4%, or 2-hour 75-gram oral glucose tolerance test (2 h-OGTT) between 7.8 and 11.1 mmol/L). HIMABERB® 500 mg was given three times daily to the treatment group, and placebo was administered three times daily to the control group. Glycemic control markers and physical parameters were evaluated for both groups on days 0, 28, 56, and 84. The glycemic control markers assessed included FPG, fasting insulin (FI), 2 h-OGTT, HbA_1c_, and homeostatic model assessment-insulin resistance (HOMA-IR). The observed outcomes were analyzed using independent t-test statistics to determine the significance of differences over time after treatment initiation and between treatment and control groups.

**Results:**

Significant decreases in all markers of glycemic control were observed in the treatment group at intermediate time points and the endpoint of the study compared to baseline levels and to the control group. For the treatment group, FPG decreased from 6.75 ± 0.23 mmol/L to 5.33 ± 0.28 mmol/L, FI from 9.81 ± 0.36 to 7.88 ± 0.52 mmol/L, 2 h-OGTT from 10.44 ± 0.52 to 8.12 ± 0.40 mmol/L, HbA_1c_ from 6.40% ± 0.20–5.43% ± 0.21%, and HOMA-IR from 3.61 ± 0.31 to 2.41 ± 0.14. The decreases in glycemic control markers compared to the control group were clinically and statistically significant (p<10^− 5^). No severe adverse effects, kidney or liver toxicity were detected.

**Conclusion:**

After 12 weeks, berberine (HIMABERB®) intervention in individuals with prediabetes significantly reduced glycemic control markers, with mean FPG and 2 h-OTGG being reduced to below prediabetic thresholds, supporting the investigation of the use of HIMABERB® for delaying progression to diabetes mellitus.

**Trial registration:**

http://ctri.nic.in**(CTRI/2021/12/038751) (20/12/2021)**.

## Introduction

Diabetes continues to be a major global health concern with a rapidly rising incidence. The incidence of conversion of prediabetes to diabetes has increased greatly. According to the International Diabetes Federation (IDF), 382 million people globally (8.3% of adults) are affected by diabetes, and the incidence is predicted to increase beyond 592 million in the next 25 years [1]. In an individual with prediabetes, the blood sugar level is higher than normal but not high enough to be diagnosed with diabetes. In the U.S., 35–38% of people are affected by prediabetes [1, 2]. According to the American Diabetes Association (ADA), an individual can be considered to have prediabetes when fasting plasma glucose (FPG) is 5.6–6.9 mmol/L, glycated hemoglobin (HbA_1c_) is 5.7−6.4%, or postprandial glucose is 140–199 mg/dl [3]. Although prediabetes does not display any physical symptoms, it nearly always precedes type 2 diabetes mellitus, which further increases the risk of microvascular diseases such as retinopathy, nephropathy, and neuropathy, along with the involvement of macrovascular systems such as heart disease and stroke [4, 5].

There is a lack of robust recommendations to prevent or delay the progression of prediabetes to type 2 diabetes because of limited pharmacological options and scarce literature on nutraceutical therapy with favorable safety profiles. ADA recommends lifestyle behavior changes to prevent the progression of prediabetes to diabetes, including weight loss, moderate-intensity physical exercise, dietary changes, and metformin pharmacotherapy [6]. Various herbs have been studied to manage symptoms due to changes in glucose metabolism, including cinnamon [7], fenugreek [8], nanocurcumin [9], tulsi [10], and soybean extract [11]. The identification of a safe, durable, and cost-effective adjunct to effectively and consistently reduce the progression from prediabetes to type 2 diabetes mellitus remains an unmet goal.

Berberine is a naturally found alkaloid with a quaternary-based chemical structure that is known to have a hypoglycemic effect. It has been used in Ayurveda in India, traditional Chinese medicine, and Middle Eastern countries for over 400 years [12]. It has recently been reported to effectively lower blood glucose and lipid levels [13, 14]. While the mechanisms of the effects of berberine on glucose are not completely known, sensitization to insulin and insulin-dependent increases in glucose consumption and uptake in adipocytes, hepatocytes and myotubes have been reported [14]. In the preventive era, efforts are increasingly focused on preventing or delaying the transition from prediabetes to diabetes mellitus [15, 16]. However, most studies on berberine have focused on type 2 diabetes mellitus patients only, with limited studies on individuals with prediabetes. The present double-blinded, randomized, and placebo-controlled trial was designed to evaluate the efficacy of HIMABERB® Berberine on glycemic control markers in otherwise healthy individuals with prediabetes.

## Materials and methods

### Preparation of HIMABERB® and placebo

*Berberis aristata* aqueous root extract was prepared by maceration at room temperature via water extraction. The extract was then concentrated and dried to achieve a purity of at least 97% berberine hydrochloride, and capsules were made with 500 mg of HIMABERB® in each capsule. Microcrystalline cellulose powder was chosen as the placebo due to its inert nature and inability to impact blood glucose markers. Opaque capsules were used for both HIMABERB® and placebo to mask the color difference and maintain the double-blind study design.

### Ethical approval

This study (approval no. SOA/IDS/IRB/2021−10) was approved by the Institutional Ethical Committee (IEC), IMS & SUM Hospital, Siksha ’O’ Anusandhan University, K−8 Kalinga Nagar. Bhubaneswar, Odisha−75,003 on May 17, 2021. The study protocol was prospectively registered at http://ctri.nic.in (CTRI/2021/12/038751) on December 20, 2021.

### Study design

The study was a randomized, double-blinded, and placebo-controlled clinical trial to evaluate the efficacy of HIMABERB® in individuals with prediabetes for 12 weeks. It was hypothesized that HIMABERB® Berberine should improve the markers of glycemic control more effectively than the placebo. After receiving a detailed explanation and providing written informed consent, 34 participants were assigned to the treatment and control groups by block randomization with parallel assignment to ensure bias reduction. Seventeen participants were assigned to each group. HIMABERB® 500 mg was given three times daily to the treatment group, and placebo was administered three times daily to the control group for 84 days. Dosing was as per the manufacturer’s instructions and after consultation with an endocrinologist. This dosing regimen is supported by previously published literature on berberine, with typical dosing of 0.5 to 1.5 g/day in trials treating diabetes mellitus [32]. Each patient kept a supplement and diet diary and a record of any adverse events on an SAE (Severe Adverse Effects) form as part of the diary. Diet and exercise routine were assessed to ensure subjects were not under any specific diet change or exercise regime. Any significant change of diet was advised to be reported and assessed at every follow up appointment. No significant change was reported for diet or exercise.

**Fig. 1:**
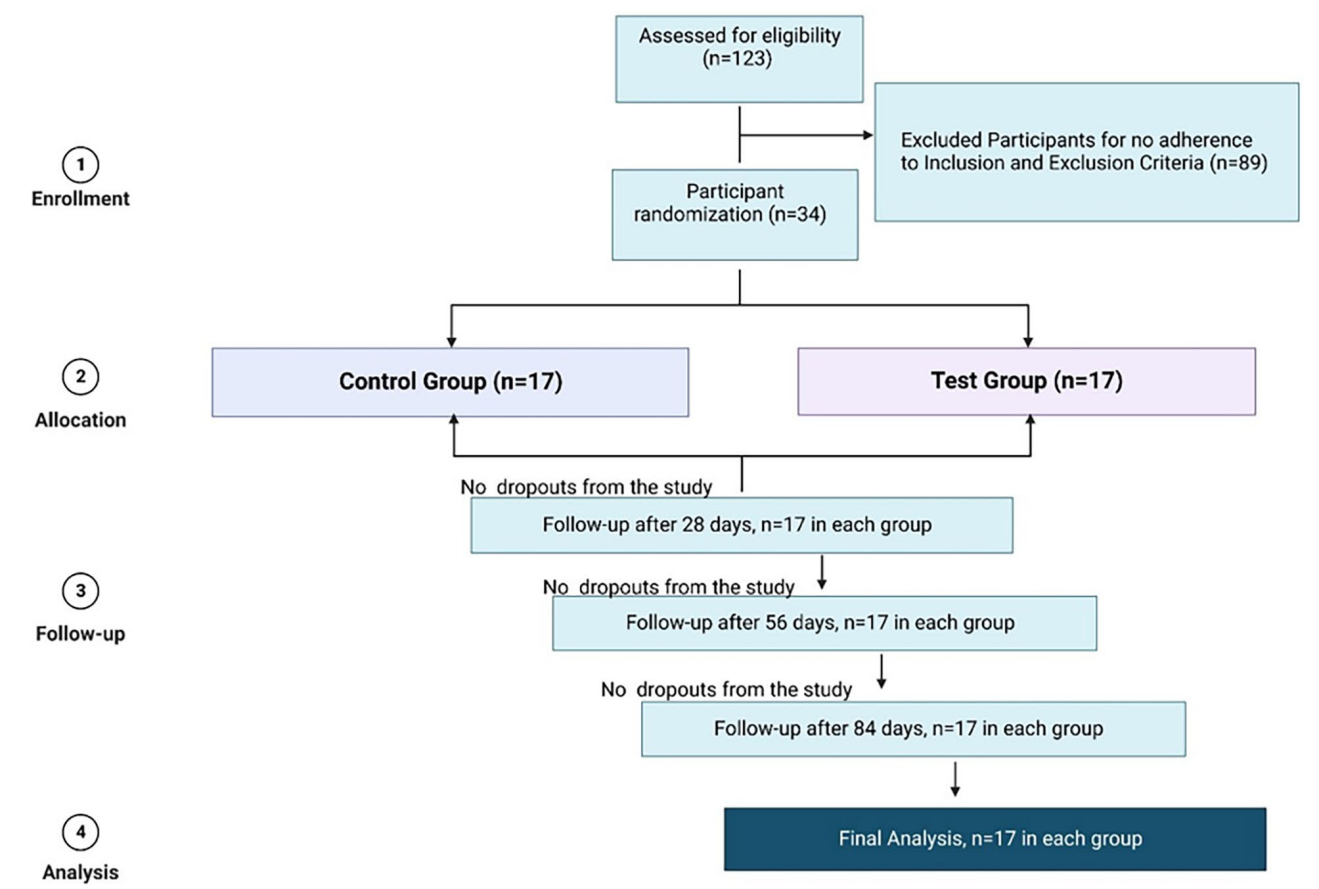
Flow chart of the study design, enrollment, randomization, follow-up, and analysis of study participants

Figure 1 shows the flow of participants through each stage of the randomized trial. The participants were screened for the clinical study based on the inclusion and exclusion criteria as detailed in Table 1, and candidates who met any exclusion criteria were eliminated. The screened participants who met all prediabetes criteria as defined by the American Diabetes Association (FPG between 5.6 and 6.9 mmol/L; HbA_1c_ between 5.7% and 6.4%; and 2-hour 75-gram oral glucose tolerance test (2 h-OGTT) between 7.8 and 11.1 mmol/L) were enrolled in the study [3]. Subjects were closed with the set inclusion and exclusion criteria as mentioned below, but were not adjusted for age sex or BMI. A total of 34 individuals were enrolled, and the study was conducted over 12 consecutive weeks (84 days). The first follow-up was scheduled on Day 28, the second follow-up on Day 56, and the third and final follow-up was scheduled for Day 84. All participants visited the study site, located at Siksha O Anusandhan (Deemed to be University), Bhubaneshwar, Odisha, 751,030, India, at baseline and at the end on the 84^th^ day. The other two follow-up visits on Days 28 and 56 were conducted at each participant’s home. At every visit, the supplement and diet diary and SAE forms were evaluated and verified. Follow up for 84 days was chosen to allow reliable evaluation of glycemic control using HbA1c over an approximately 3-month period.

**Table 1:** Inclusion and exclusion criteria for the study

**INCLUSION CRITERIA**
1. Age 18 to 55 years 2. Male or female gender 3. Females of childbearing age to agree to use birth control methods during the trial period and show a negative pregnancy test at the time of recruitment 4. Fulfillment of the prediabetic diagnostic criteria as per the American Diabetes Association, taken within 12 weeks. (FPG between 5.6 and 6.9 mmol/L; HbA_1c_ between 5.7% and 6.4%; and 2 h-OGTT between 7.8 and 11.1 mmol/L) 5. Agreement to continue the current diet and refrain from any new supplements 6. Ability to read and understand English 7. Agreement to provide informed consent to the trial with the ability to understand the risks and benefits of the protocol 8. Willingness to complete intake form, supplement and diet diary, and records associated with the study 9. Willingness to cooperate for follow-up calls and visits
**EXCLUSION CRITERIA**
1. Pregnant or lactating women, or women planning to become pregnant in the next 12 weeks 2. Transgender individuals or individuals taking a hormonal injection 3. Comorbidities including malnourishment, impaired hepatic or renal function, cardiac diseases, any acute/severe diseases, or having undergone bariatric surgery 4. Currently taking weight loss medications, any oral hypoglycemic medication, insulin injection, or steroids 5. Current daily use of herbal/nonherbal supplements 6. Daily tobacco use, or individuals with any substance abuse issues in the past or present 7. Known allergies to any substances in the product 8. Any significant neurological and/or psychiatric conditions

### Observation indicators

Preliminary screening and assessment were carried out for every individual to collect medical history, physical examination results, blood pressure, heart rate, body weight, waistline, liver function, kidney function, fasting plasma glucose (FPG), fasting insulin (FI), 2-hour oral glucose tolerance test (2 h-OGTT), glycosylated hemoglobin (HbA_1c_), and homeostatic model assessment-insulin resistance (HOMA-IR). Participants fasted for at least 8 h before blood samples were collected at approximately 0800 h, and samples were tested using an automatic biochemical analyzer (Cobas Integra 400 Roche, make−2018, Germany). The Cobas HbA_1C_ test used is certified by the National Glycohemoglobin Standardization Program according to the manufacturer’s information. All parameters were recorded at every follow-up visit. HOMA-IR was calculated by Eq. (1):

1 $$HOMA-IR=\frac{\left(FIxFPG\right)}{22.5}$$

### Safety assessment

Participants were interviewed regarding side effects and adverse reactions during the follow-up visits. SAE forms were part of the patient diary, which were explained and reinforced at every follow up visit and on telephone calls once a week. The supplement and diet diary were checked at each follow-up to ensure completion and uniformity. To evaluate liver and kidney function and ensure safety for individuals in the treatment group, aspartate aminotransferase (AST), alanine aminotransferase (ALT), alkaline phosphatase (ALP), and blood urea nitrogen to serum creatinine ratio (SC) levels were measured at the start of the study, 28 days, and 84 days. As the follow-up duration was limited to 84 days, SC ratios were used to quantifiably assess short-term renal toxicity for statistical analysis. Participants were routinely reminded to maintain the supplementation regimen and to update the supplement and diet diary regularly.

### Statistical methods

Data collected on the randomized participants were tabulated as per the guidelines of CONSORT (Consolidated Standards of Reporting Trials). The statistical analysis was performed using GraphPad Prism (Version 9.3.1, GraphPad Software San Diego, CA 92,108) software for Windows computers. Descriptive analysis and independent t-tests were performed for the obtained tabulated values with statistical significance set at *p <* 0.05. Data are presented as the mean ± SD. Sample size was determined to achieve statistical power of 80% and a significance level of < 0.05 using two-factor ANOVA without replication for analyses within (time points) and between groups (control versus treatment). For categorical data (gender) chi square test was used. Anticipating a difference in means of 0.91 SD and 10% attrition, a total sample size of 34 was derived. Time series data were analyzed using Pearson’s correlation test and comparisons of correlations between treatment and control groups were done using one-sided Fisher’s Z-Test.

## Results

### Participant characteristics

A total of 34 individuals with prediabetes fulfilling the inclusion and exclusion criteria were enrolled (n = 17 in each group). Baseline features were matched for the treatment and control groups (Table 2**)**. Adherence to the supplement regimen was found to be equivalent in the treatment and control groups.

**Table 2:** Baseline demographic characteristics (mean ± SD)

Variables	Treatment Group (n = 17)	Control Group (n = 17)	p-value*
**Age (years)**	43.5 ± 8.9	44.8 ± 8.6	0.67
**Male: Female**	1.4:1	1.1:1	0.62
**Weight (kg)**	78.7 ± 19.1	80.0 ± 16.4	0.84
**Waistline (cm)**	85.8 ± 7.9	85.9 ± 8.9	0.96
**Hipline (cm)**	93.6 ± 9.5	92.8 ± 9.5	0.76
**BMI (** ^**kg/m2**^ **)**	25.6 ± 3.3	25.4 ± 5.0	0.91

BMI: Body Mass Index *two-way ANOVA without replication for qualitative data and chi-square for categorical data (gender)

### Efficacy

**Fig. 2:**
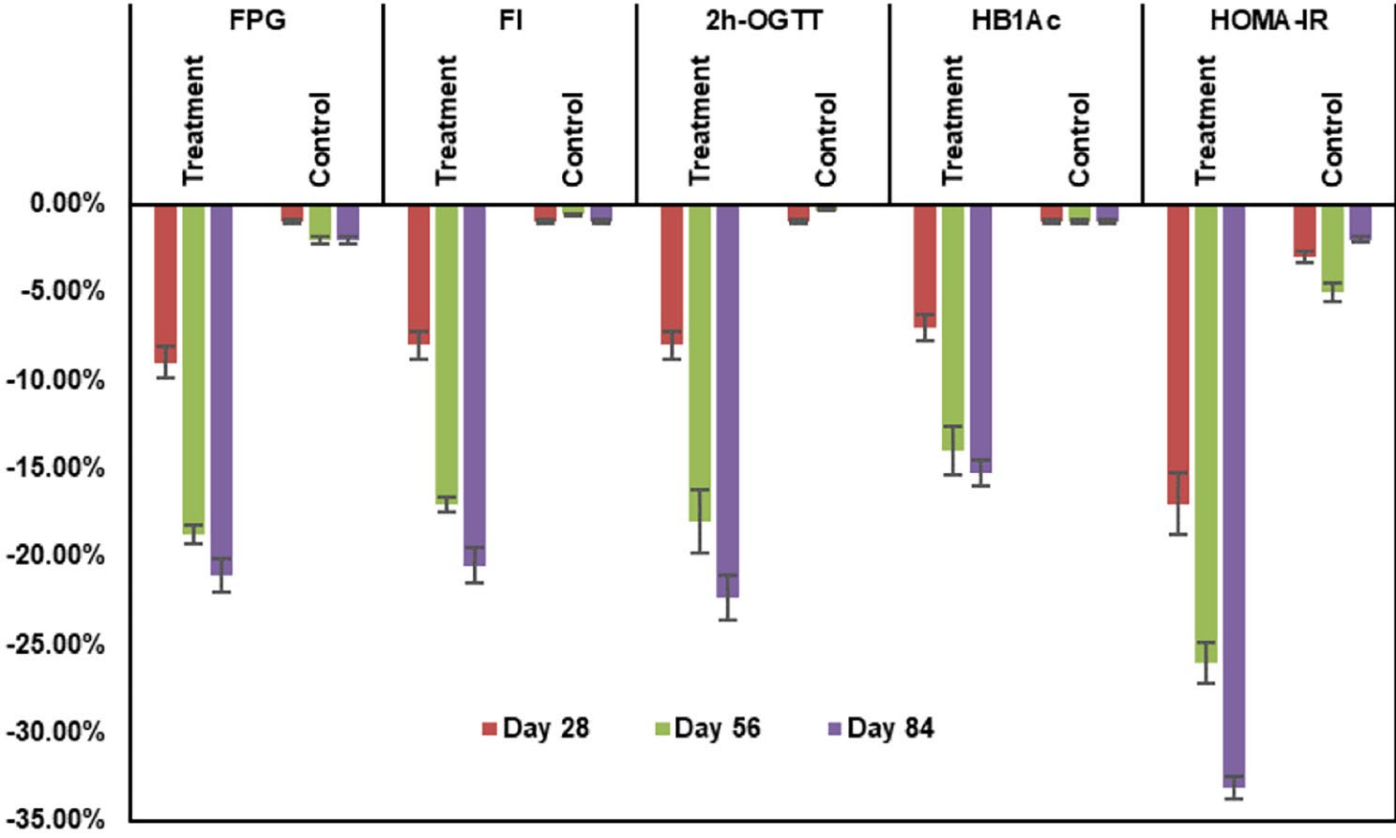
Changes in glycemic control markers (mean ± SD) in the treatment and control groups at different time points across FPG (mmol/L), FI (µIU/mL), 2 h-OGTT (mmol/L), HbA_1c_ (%), and HOMA-IR (units). FPG: Fasting Plasma Glucose; FI: Fasting Insulin; 2 h-OGTT: 2-hour Oral Glucose Tolerance Test; HBA1c: Hemoglobin A1c; HOMA-IR: homeostatic model assessment-insulin resistance. Error bars indicate one standard deviation from the mean. *ANOVA p < 10 ^− 5^ versus control

A detailed analysis of the treatment group at different time points revealed that the glucose marker values consistently declined over time (Fig. 2).

All measured glycemic control markers showed a significant reduction over time in the treatment group. From t_0_ to t_3_, in 84 days, FPG values saw a total 21.01% reduction in mean values, FI saw 19.68% reduction in mean values, 2 h-OGTT mean values were down by 22.15%, HbA_1c_ mean values were down by 15.17% and HOMA-IR units declined by 33.39% in mean values (ANOVA *p <* 0.10^− 5^). There was also a consistent decline in all parameters from baseline (t_0_), to 28 days (t_1_), 56 days (t_2_), and 84 days (t_3_). No measures were significantly decreased in the placebo group at any time point. Comparisons of Pearson’s correlation coefficients between control and treatment groups showed significant differences in correlations over time with p-values of < 0.005 for all measures.

FPG after 84 days was significantly lower in the treatment group than in the control group, with a mean FPG of 5.33 ± 0.28 mmol/L for the treatment group and 6.16 ± 0.44 mmol/L for the control group (*p<*10^− 5^). FI after 84 days was also significantly lower in the treatment group than in the control group, with a mean FI of 7.88 ± 0.52 µIU/mL for the treatment group and 9.76 ± 0.37 µIU/mL for the control group (*p<*10^− 5^). Similar significant reductions were seen after 84 days in 2 h-OGTT, with a mean value in the treatment group of 8.12 ± 0.40 mmol/L which was significantly lower than the control group with a mean value of 9.68 ± 0.51 mmol/L (*p<*10^− 5^). HbA_1c_ values after 84 days were also significantly lower for the treatment group at 5.43 ± 0.21 as compared to the control group with mean values at 6.10 ± 0.24 (*p<*10^− 5^). HOMA-IR showed a similar significant reduction for the treatment group with mean values after 84 days at 2.41 ± 0.14 units compared to the control group mean values at 3.40 ± 0.28 (*p<*10^− 5^), as shown in Table 3. The treated group showed statistically significant reductions in these values at all three time points, 28, 56, and 84 days, compared to baseline values, and all glycemic measures were found to be significantly lower in the treatment group when compared to the control group (*p<*10^− 5^).

**Table 3:** Changes in FPG, FI, 2 h-OGTT, HbA_1c,_ and HOMA-IR after intervention (at Day 84) in the treatment and control groups, mean ± SD (Significance level ANOVA *p* value < 10^− 5^)

Parameters	Treatment Group (n = 17)	Treatment Group (n = 17)	Control Group (n = 17)	Control Group (n = 17)	*p* value Treatment vs. Control
	Day 0	Day 84	Day 0	Day 84	Day 84
FPG (mmol/L)	6.75 ± 0.23	5.33 ± 0.28*¥	6.31 ± 0.44	6.16 ± 0.44	*p<10* ^*− 5*^
FI (µIU/mL)	9.81 ± 0.36	7.88 ± 0.52*	9.85 ± 0.35	9.76 ± 0.37	*p < 10* ^*− 5*^
2 h-OGTT (mmol/L)	10.44 ± 0.52	8.12 ± 0.40*	9.71 ± 0.63	9.68 ± 0.51	*p < 10* ^*− 5*^
HbA1_c_ (%)	6.40 ± 0.20	5.43 ± 0.21*¥	6.15 ± 0.26	6.10 ± 0.24	*p < 10* ^*− 5*^
HOMA-IR (units)	3.61 ± 0.31	2.41 ± 0.14*	3.45 ± 0.26	3.40 ± 0.28	*p < 10* ^*− 5*^

FPG: Fasting Plasma Glucose; FI: Fasting Insulin; 2 h-OGTT: 2-hour Oral Glucose Tolerance Test; HBA1c: Hemoglobin A1c; HOMA-IR: homeostatic model assessment-insulin resistance. All values represent means. *ANOVA p-values for differences between day 84 and day 0<10^− 5^. ¥ below prediabetic threshold

### Safety

The safety parameters (AST, ALT, ALP, and SC levels) were checked for the treatment group at baseline, after the first follow-up at 28 ± 2 days, and after the last follow-up at 84 ± 2 days. All treatment group values remained within normal levels (Table 4). No severe adverse events were reported on SAE forms or in interviews. Three individuals in the treatment group self-reported mild nausea or vomiting in the first week of intervention, which was verified at the first follow-up at 28 ± 2 days. All three cases were self-limiting and did not cause any participant to drop out of the study.

**Table 4:** Changes in the safety parameters ALP, AST, ALT, and SC at baseline (at Day 0) and after intervention (at Day 84) in the treatment group, mean ± SD

Parameters	Treatment Group (n = 17)	Treatment Group (n = 17)	p-value*
	Day 0	Day 84	
ALP (U/L)	79.00 ± 2.18	73.65 ± 5.35	0.00014
AST (U/L)	30.00 ± 1.73	27.82 ± 2.60	0.00011
ALT (U/L)	31.41 ± 1.58	29.41 ± 3.52	0.0013
SC (mg/dL)*	17.94 ± 2.16	17.47 ± 2.00	0.15

*Measured as Blood Urea Nitrogen to Serum Creatinine Ratio. ALP: Alkaline Phosphatase; AST: Aspartate Aminotransferase; ALT: Alanine Transaminase; SC Serum Creatinine *two-way ANOVA

## Discussion

Individuals with prediabetes are at higher risk of progressing to type 2 diabetes mellitus and resulting cardiovascular complications such as myocardial infarction and cardiovascular death. They are more prone to nephropathy, neuropathy, and retinopathy as a result of diabetic comorbidities [17, 18].

Dietary supplements are commonly used by a large population around the globe for a variety of health-related goals. In the case of individuals with prediabetes, glycemic control and lifestyle modifications remain the mainstay in preventing and delaying its progression to diabetes. With limited proven pharmacological agents, dietary supplementation is increasingly being considered an early intervention for the prevention of prediabetes [19].

Berberine (BBR, molecular formula: C_20_H_19_NO_5_, molecular weight: 353.36), a natural extract of *Coptis chinensis*, *Berberis aristata*, and Phellodendron bark, has been used for the symptoms of diabetes [20]. Various in vivo studies have shown promising improvements in diabetes treatment [21]. Berberine-mediated activation of protein kinase (AMP-activated protein kinase) is partially responsible for its effects on glycemic control, stimulates glucose and fatty acid oxidation in cells, and enhances insulin sensitivity [22, 23].

The present study was conducted to explore the efficacy of HIMABERB® on glycemic control markers in otherwise healthy individuals with prediabetes. In the present study, HOMA-IR was also significantly reduced in the treatment group. The effects observed in our study were significantly higher than those in a previous study, which may be attributable to a higher dosage in our study of 500 mg three times daily versus 300 mg three times daily [24]. The results of our study are consistent with the results obtained by Wang et al. in 2020, which demonstrated a significant reduction in fasting glucose, 2 h-OGTT, and HbA_1c_ in individuals with prediabetes [24]. A consistent decline in glycemic markers was observed over time, with no deviation of safety parameters outside the normal range. HIMABERB ® Berberine 500 mg administered three times daily for 12 weeks to patients with prediabetes was safe and effective in decreasing glycemic control markers (Fig. 2; Table 4).

In a meta-analysis and review of 17 randomized controlled trials on berberine for the treatment of diabetes mellitus, Wei XC et al. [13] concluded that berberine significantly reduces glycemic control markers, including fasting plasma glucose, postprandial blood glucose, and HOMA-IR, in comparison to a control group of placebo or no intervention with medicine [14, 22, 25–29]. The results of this double-blind, randomized, placebo-controlled trial demonstrate the effectiveness of HIMABERB® in prediabetes and are in alignment with previous studies on diabetic individuals.

In 2019, Friedman et al. conducted a study to assess the effect of polyherbal supplements on prediabetic adults. The supplement was a combination of cinnamon bark, banana leaf, kudzu root, fenugreek seed, gymnema leaf, and berberine hydrochloride. The researchers concluded that herbal supplements, in the combination above, can be used as an adjunct in preventing progression to type 2 diabetes mellitus [30, 31].

The intervention used in the present study contained only HIMABERB® (a high purity and water-extracted berberine hydrochloride) without any excipients. Strict adherence to the protocol was maintained in this double-blinded, randomized, placebo-controlled trial, with regular follow-ups on participants’ supplements and diet diaries. Berberine has been associated with gastrointestinal discomfort and adverse events, including nausea, vomiting, diarrhea, constipation, and abdominal discomfort [32]. Consistent with these findings, three mild cases of nausea or vomiting were seen in this study. No clinically relevant changes were seen in vital signs or levels of AST, ALP, ALT, or SC, which remained within normal limits, indicating that the supplement is safe and well tolerated (Table 4).

Treatment with HIMABERB® over the course of 84 days resulted in decreases in mean FPG and HbA1c to below the clinically defined thresholds for prediabetes. These results are clinically meaningful and have the potential to impact the management of patients that are classified as prediabetic. Control of blood glucose in individuals with prediabetes to below clinical thresholds using natural and non-toxic agents, as demonstrated here, may benefit disease outcomes and safety for patients.

Given its potential insulin sensitizing effects as discussed above [14], berberine may be useful in intervention for early stages of insulin resistance in patients who have not been diagnosed with diabetes. In addition, given its favorable safety profile and evidence of efficacy, berberine may be an attractive supplemental therapy for glycemic control compared to other supplemental drugs with less conclusive evidence for safety and efficacy, such as chromium, magnesium, nicotinamide or vanadium.

While statistically and clinically significant effects of HIMABERB® on glycemic markers in patients with prediabetes were clear, this study was limited by a small sample size, follow up that was limited to 84 days, single-institution design, and lack of analysis of durability and dose-dependency. Additionally, data were not adjusted for age, sex or BMI, which may have confounding effects on analyses. A multicentric long-term study with a larger sample size and adjustments may provide more robust data that is generalizable to the broader population.

## Conclusion

Intervention with HIMABERB® in individuals with prediabetes for 84 days (12 weeks) significantly affected glycemic control markers, supporting the investigation of its use to delay progression to diabetes mellitus. The results of this study suggest that treatment is safe and effective in controlling glycemic control markers in patients with prediabetes. Further large multicenter trials are warranted.

## Data Availability

The datasets during and/or analysed during the current study available from the corresponding author on reasonable request.
